# Comparison between different surface treatment methods on shear bond strength of zirconia (*in vitro* study)

**DOI:** 10.4317/jced.56242

**Published:** 2020-03-01

**Authors:** Christelle Joukhadar, Essam Osman, Mohammad Rayyan, Mohammed Shrebaty

**Affiliations:** 1 Clinical Instructor at Faculty of Dentistry, Beirut Arab University, Beirut, Lebanon; 2 Professor of Dental Materials, Faculty of Dentistry, Beirut Arab University, Beirut, Lebanon; 3 Professor of Prosthodontics, Faculty of Dentistry, Beirut Arab University, Beirut, Lebanon; 4 MSC in Fixed prosthodontics, Faculty of Dentistry, Beirut Arab University. Beirut, Lebanon

## Abstract

**Background:**

To compare the effect of Er:YAG Laser and Air particle abrasion (APA) surface treatments on shear bond strength of Y-TZP to composite resin cuboids in the presence and absence of primer application and salivary contamination.

**Material and Methods:**

Seventy-two cuboidal shaped specimens 7x7x3 were prepared from Y-TZP using CADCAM, cleaned and sintered. Specimens were divided into 2 main groups (n=36) according to surface treatment method; Air particle abrasion (A) and laser (L). Each group was subdivided into 2 subgroups (N = 18) according to surface modification using primer; each subgroup was further divided into 2 subdivisions (N=9) according to the presence of salivary contamination; APC (Air particle abrasion, primer, contamination), AP (Air particle abrasion, primer), AC (Air particle abrasion, contamination), A (Air particle abrasion), LPC (Laser, primer, contamination), LP (Laser, primer), LC (Laser, contamination), L (Laser). Composite cuboids having dimensions of 6x6x3 were also fabricated using custom made plexi plates. Composite cuboids were cemented centrally to zirconia cuboids and light cured under 5 kg weight for 6 mins. Shear bond strength of specimens was measured utilizing universal testing machine at a crosshead speed of 0.5 mm/min. Failure loads were recorded in Newton. SBS was calculated according to equation: SBS (MPa) = load (N)/area(mm2).

**Results:**

Viewing shear bond strength between studied groups, group APNC (484.02±85.02) showed higher mean value compared to ANPNC (122.09±55.80), also LNPNC (120.87±65.10) showed higher mean value in comparison to LPNC (170.78±53.22). APNC (484.02±85.02) and APC (592.22±189.65) showed higher mean values than LPNC (170.78±53.22) and LPC (3227.66±108.28) in sequence.

**Conclusions:**

APA showed higher SBS values than Er:YAG surface treatment. Primer showed better results than no primer coating. Artificial saliva contamination did not affect the SBS of zirconia compared with no contamination results.

** Key words:**Shear bond strength, zirconia, air particle abrasion, Er:YAG laser, primer, contamination.

## Introduction

Ceramics have received much praise and clinical success since their introduction, because of their ability to mimic color and translucency of natural teeth. This urges manufacturers to improve weak points inherited in ceramics, which included brittleness and low tensile strength. This led to marked increase in the use of metal free restorations.

Despite its mechanical strength, zirconia was accused of having poor bond strength to cement and veneer material. Unfortunately, establishment of a durable chemical or mechanical bond to zirconia has been proven to be difficult because of its surface stability (1), which required different surface treatment methods than those adopted with glass-ceramics.

It goes without saying that; the clinical success of bonded ceramic restorations depends on the cementation process. There is a general agreement that air particle abrasion (APA) using 50–110 µm alumina particles at 0.25 PSI is effective in cleaning and roughening the bonding surface of zirconia ceramic (2). Furthermore, clinical experience with primer has indicated improved bonding (3), additionally; the surface should be free from any contaminants to prevent the decrease in bonding efficacy (4).

Recently, laser pretreatment is suggested to change surface of materials aiming to improve the bonding to dental structure. Some studies have suggested application of lasers to change zirconia surface in order to improve their bond to tooth structures. Whereas other studies, demonstrated that in comparison to conventional zirconia surface treatment methods; laser pretreatment was not efficient for increasing bond strength or even decreasing it (5).

This raises another major issue, jeopardizing optimum ZrO2- resin bond related to potential contamination before cementation. After APA and clinical try-in procedures, contamination can’t be avoided by blood, saliva or silicone indicator. This may lead to compromised bonding protocol and questionable bond strength (6). To complicate matters more, zirconium shows a strong affinity towards the phosphate group found in saliva and other fluids, which reacts with the zirconia surface and makes bonding difficult by reducing resin bond strength (7).

Several methods were suggested to prevent contamination after try-in stage, the most commonly used, was application of primer to the APA surface prior to try-in step, which was found to be time saving method by eliminating the need of additional laboratory step to repeat

APA to the intaglio surface and cause further delay of cementation (8).

## Material and Methods

-Specimen grouping

Total sample size was 72 cuboids divided into two main groups (N=36) according to surface treatment (Air particle abrasion (A) and laser (L)). Each group was further subdivided into two subgroups (N = 18) according to surface modification using primer. The two subgroups were further divided into two subdivisions (N=9), according to the presence of salivary contamination (Table 1).

**Table 1:**
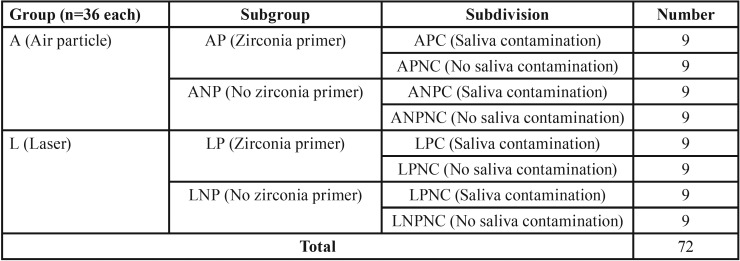
Specimens grouping.

-Fabrication of Zirconia ceramic specimens

**Figure 1:**
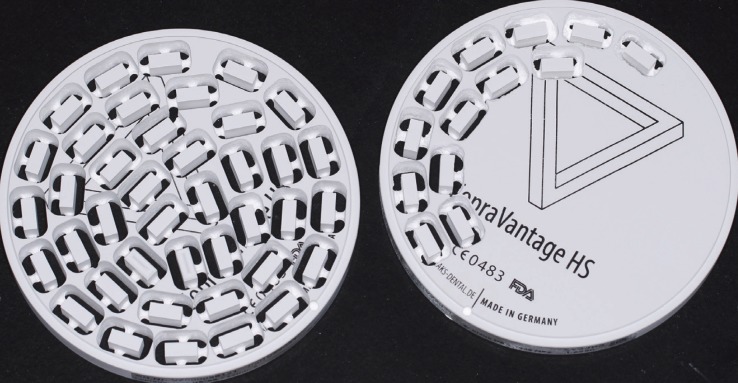
Milled zirconia cuboids.

Using AUTOCAD (Autodesk, mac, 2017), a cuboidal shape of 7x7mm dimension and thickness of 3mm was drawn and imported as STL (Standard Triangulation Language) file to CAD/CAM software (Rainbow mill, Seoul-South Korea). A block of each material was mounted in the milling machine in dry mode using burs that are specially developed for milling dental zirconia (Fig. 1). Accordingly, 72 cuboids were milled and then cleaned using jets of air and ultrasonic solution for 15 sec (9). Zirconia cuboids were left to dry, sintered according to manufacturer direction, then randomly divided among the groups.

-Fabrication of Resin blocks 

Transparent plexi-frames of 3mm thickness were prepared by laser cutting. In the middle of this frame, square mould of (6x6) was laser cut in order to standardize the size of resin cuboids obtained from plexi. Another transparent plexi-frame was cut to act as floor. The 2 plexis were placed above each other. SDR composite was then injected into cuboidal mold created in the middle of plexi. Histology glass slide was placed onto to ensure flat composite surface which was then light cured. After curing, 2 plexis were separated from each other and composite was pushed from cuboidal space.

-Surface Treatment

• Air Particle Abrasion

**Figure 2:**
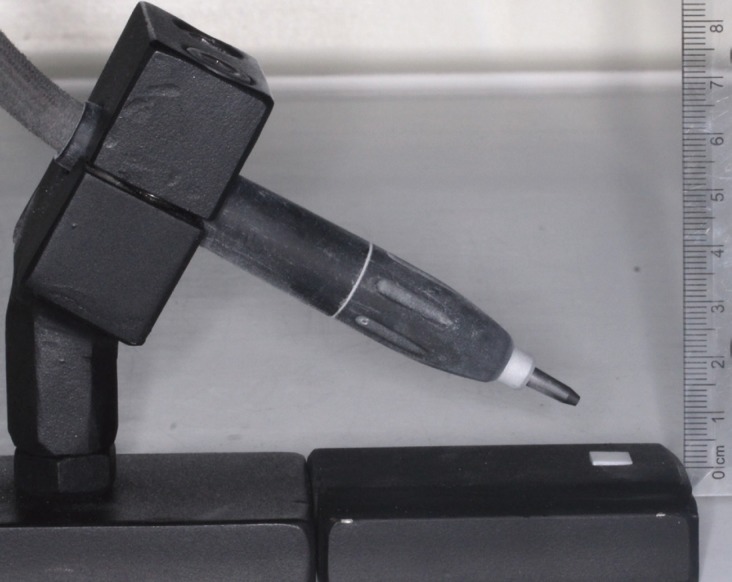
Custom made laboratory jig for APA surface treatment.

Jig was constructed to standardize 60 degrees angle and 1cm distance between application tip and zirconia surface for group (A) specimens (Fig. 2). Zirconia holder was fabricated to secure zirconia cuboids during APA. It has hole in the middle having same dimensions of zirconia cuboid allowing them to be flushed with its surface during APA. Holder moves freely to allow brushing application of APA. Zirconia specimens were APA with Al2O3 (50µm) at pressure 2 Bar for 20 sec in a brushing motion (10).

•Laser surface treatment.

Er:YAG laser was applied to group L with special handpiece. Zirconia holder was constructed to maintain laser tip perpendicular to ceramic surface at a distance of 1 mm, and restricted ceramic area was laser treated with water irrigation and air cooling for 15 sec. Table 2. presents laser parameters.

**Table 2:**
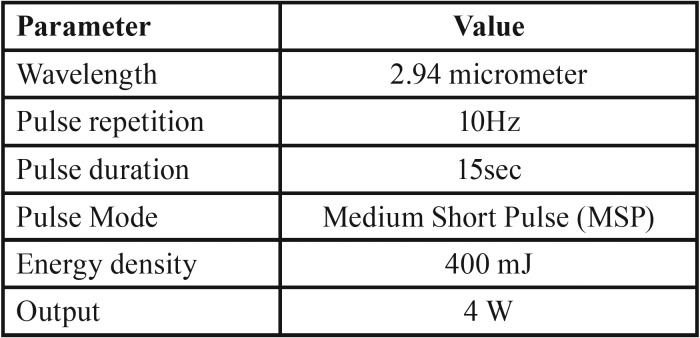
Laser parameters.

-Application of zirconia primer.

Zirconia primer was applied to zirconia surface using clean disposable brush and was left for 20 sec and then thinned out by air.

-Saliva contamination of specimens.

Saliva substitute was prepared composed of (Na2HPO4: 0,426%, NAHCO3: 1,68g, CaCl2: 0,147 g , H2O: 800 ml, HCL-1M: 2,5ml ) (11). Each subdivision was placed solely in solution to avoid mixing. Solution was refreshed after each subdivision. Specimens, were immerged in saliva substitute at 37°C for 1min, then specimens were rinsed with tap water for 15 sec and air-dried for 15 sec (4).

-Cementation of Zirconia Cuboids to Composite cuboids.

**Figure 3:**
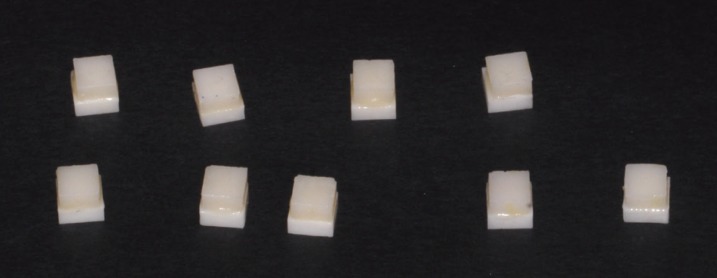
Cemented specimens.

Two transparent 3mm thick plexi frames of the same size were prepared by laser cut. One transparent plexi frame was laser cut with a space of (7.2x7.2) in middle to facilitate placement of zirconia into the plexi without touching it. Second one was also laser cut with (6.2x6.2) space in middle. Bond was applied on intaglio surface of composite cuboids then self-adhesive resin cement was auto-mixed and applied surface (primed or not, contaminated or not). Finally, zirconia specimens were placed over composite resin specimens under 5 kg weight for 6 min and light cured. Fig. 3. showed all specimens were cemented in the same manner.

-Specimen testing

A framework to hold the specimens was fabricated. The plexi was laser cut with a dimension of 7x6x7 and cemented to another plexi to act as a floor. Chisel tip was applied vertically to the cement zirconia interface at cross head speed of 0.5mm/min until failure which was defined by sudden drop of load (in newton). SBS was calculated according to equation: SBS (MPa) = load (N)/area(mm2).

-Statistical analysis

Kolmogorov-Smirnov normality tests was considered to evaluate normality data distributions. Independent T-student tests were conducted to analyze differences in shear bond strength according to surface treatment method, primer coating, and saliva contamination.

All statistical analysis was conducted using SPSS v.17 (BM Corp; Armonk, NY). Charts were created using Microsoft Excel 2018. An alpha level of 0.05 was used as a decision point for statistical significance.

## Results

Effect of saliva contamination on SBS values (MPa) according to primer coating and surface treatment:

According to independent T student test results in Table 3, SBS values of air particle abrasion group APC showed highest SBS mean value (592.22), followed by APNC (484.0), ANPC (273.50) and ANPNC showed lowest mean value (122.09). On the other hand, laser surface treatment, LPC (324.66) showed highest mean values, followed by LPNC (170.78), LNPNC (120.87) and LNPC showed lowest mean value (111.02).

**Table 3:**
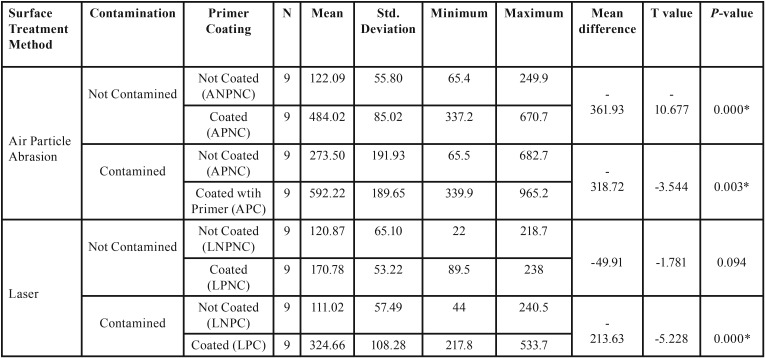
SBS values (MPa) according to surface treatment method, primer coating, and contamination with saliva.

## Discussion

A successful restoration is not only defined by its strength, but mainly by stability of the cement-restoration adhesion. Zirconia is widely applied in the dental field due to its strength, esthetics and biocompatibility. Unfortunately, debonding of zirconia restorations has been observed in clinic situations. Many studies were carried out to evaluate the best surface treatment protocol.

In present study, laser parameters were previously tested by Subaşı and İnan, they found to be effective and produced roughed surfaces (12). According to Cavalcanti *et al.*, reported acceptable surface roughness by Er:YAG laser at 400 and 600 mJ pulse energy (13). These results were contradictory with those found by Akyil *et al.*, they showed that Er:YAG can be beneficial at 2 W power and 200 mJ pulse energy as well; irradiation time for this setting was 10 seconds (14). Furthermore, Arami *et al.* (2014), they compared effect of different Er:YAG output power (1.5, 2 and 2.5) with different lasers and particle abrasion on surface characteristics of zirconia ceramics, they concluded that treated surfaces by Er:YAG laser and air abrasion showed similar surface roughness (15).

APA surface treatment creates micro-irregularities on sintered zirconia, which improves SBS. In current study,APA surface treatment using Al2O3 (50 μm) showed highest SBS, similar results were found by Özcan *et al.* evaluated effect of air-particle abrasion protocols on zirconia specimens, they studied effect of 50μm Al2O3 and other particle sizes on surface roughness, they concluded that APA zirconia by 50μm Al2O3 is capable of producing more roughness when compared to other particle size (16). Skienhe *et al.*, studie effect of different types of abrasive surface treatment before and after zirconia sintering, they found that highest significant shear bond strength value when APA 50 μm Al2O3 was applied after sintering (17). These findings were opposite to earlier results stated by Hallmann *et al.*, they studied effect of blasting pressure, abrasive particle size and grade, on phase transformation and morphological change of dental zirconia surface, their results showed that abrasion of ceramic surface with 50 or 110 μm alumina airborne particle at pressures of 2.5 or 1.5 bar, respectively, was regarded as the optimum blasting condition (18). Su *et al.*, evaluated effect of various sandblasting conditions on surface changes of dental zirconia, according to their results, they recommended for dental applications to use APA with Al2O3 particles at 0.2 MPa, 21 sec and the powder size of 110 µm to improve bonding (10).

In current study, results showed that primer coating after surface treatment, increases SBS, even in the presence of saliva contamination. In previous studies, it was concluded that primer application on zirconia treated surface will protect the surface treatment from any contamination, assure good strength. That’s why in current study, primer was used with and without contamination, to check its efficacy in protecting treated surface (4).

Additionally, surface should be free from any contaminants to prevent decrease in bonding efficacy (7). Hence, in clinical try-in stage contamination can’t be avoided by blood, saliva, and water or silicone indicator. This may lead to compromised bonding protocol (6). Artificial saliva contains only inorganic components, such as calcium and phosphate, and does not contain human salivary proteins. However, use of human saliva in experimental studies may lead to problems in reproducibility and standardization of experiments due to human variation. Consequently, artificial saliva was used in this study for standardization, with or without zirconia primer, to identify if primer protects treated surface from contamination, in addition to the effect of contamination in decreasing bonding effect. However, results obtained using artificial saliva may differ from those obtained using human saliva. Moreover, self-etch resin cement was used in this study to decrease cementation steps and reduce error. As well, cementing plexi frames were fabricated to perfectly cement all specimens in exact position. Up to authors knowledge, no studies were done to compare effect of APA and laser surface treatment on SBS of zirconia, in case of primer application and artificial salivary contamination of surface.

In this *in vitro* study, results showed that APA effect on shear bond strength was higher than laser surface treatment. Demir and Ozturk *et al.* and Caglar *et al.*, demonstrated that APA method provided rougher surfaces than Er:YAG laser radiation and this method can be used to obtain micromechanical retention (19).

Turp *et al.*, concluded that whatever the size of Al2O3 particles and APA time, it would increase the possibility of phase change and creation of surface roughness increases (20). In current study, highest shear bond strength mean value recorded for sandblasting could be attributed to increase in surface area created by APA allowing accepTable roughness facilitating resin-ceramic micromechanical interlocks formation. It was also suggested that APA reduces inherent surface defects or those generated as a result of manufacturing process. In contrast with the results of some previous studies, Akin *et al.* and Dede *et al.*, concluded that Er:YAG laser treatment increased bond strength of zirconia compared to APA treatment (21,22).

In present study, it could be suggested that primer coating is effective in increasing bonding of zirconia with or without contamination, when used after APA surface treatment. Results of current study were in accordance with Cavalcanti *et al.*, who investigated bond strength of resin cements to a zirconia ceramic with different surface treatments, their results showed that air abrasion with Al2O3 (50μm) when associated with a primer gave the highest bond strength (13). Moreover, Gargari *et al.* conducted a literature review on cementation of zirconia, they concluded that zirconia should be treated with Al2O3 (50μm) and cemented with resin containing MDP which is considered best standard in enhancing and increasing bond strength (23). Moreover, Attia and Kern and Zhang *et al.*, evaluated effect of primer on bond strength and durability of zirconia and concluded that primer could improve the primary resin bond strengths of zirconia ceramics (24).

The challenge in promoting a strong, reliable bond between the intaglio surface of zirconia and tooth structure, lies in achieving surface free of the contaminants that often result from intraoral try-in procedures. In this study, surprising results revealed that the contaminated surface of zirconia yielded better result than the contamination free surface when treated with APA and coated with primer. These results may be explained by the fact that some of the artificial saliva inorganic components left after water washing, could had bound to specific primer element that led to increase the SBS of Y-TZP. Furthermore, these results could be by the effect of primer on zirconia surface, by making it more hydrophobic, leading to decrease effect of contamination on treated surfaces.

Stated by Angkasith *et al.*, they concluded that when primer is applied prior to salivary contamination, water spray is able to return bond strength to its original state, according to their results, application of primer made the zirconia surface slightly more hydrophobic, probably, reducing salivary wetting ability and deposition of organic residue (8). Krifka *et al.*, studied effect of decontamination and cleaning on shear bond strength of Y-TZP, they concluded that, coating with primer containing 10-MDP prior to human saliva contamination and followed by water preserved bond strength, suggesting that the zirconia surface is somewhat saturated and that, interaction of phosphate compounds from saliva is impossible (25). Nagaoka *et al.*, studied chemical interaction mechanism of 10-MDP with zirconia, according to their findings, presence of phosphate groups was confirmed originating from primer containing 10-MDP at zirconia surface, even after washing with acetone, thereby indicating that strong interaction of primer with zirconia resisted washing (26). Nevertheless, Aladag *et al.*, concluded that, cementation surface contamination of zirconia restoration and inadequate removal of contaminants increase risk of failure (27). Yoshida, stated that saliva contamination significantly reduced bond strength of resin cement to zirconia (28). Clearly similar results could not be expected when clinical contamination of Y-TZP by natural saliva occurs due to the difference in composition.

Earlier studies, stated that, retreating the zirconia surface prior to cementation, will ensure good bonding results. Amaral *et al.* stated that, abrasive process removes loose contaminated layers, increases surface area available for bonding and improves wettability of luting material. Yang *et al.*, have suggested that an additional particle abrasion may give good bonding results after contamination, comparable to that seen in groups without contamination (4). Moreover, Chintapalli *et al.*, stated that use of a second particle abrasion could be controversial as a result of potentially deleterious effect on zirconia phase transformation that could possibly weaken the zirconia ceramic (29).

Shear bond strength showed higher results when zirconia is treated with APA, laser surface treatment showed lower results. Treating zirconia surface with primer significantly increases shear bond strength of zirconia, it is recommended to coat zirconia surface using primer after treating with APA for better bonding. Artificial saliva significantly increased shear bond strength of resin to zirconia when used after APA or Laser surface treatment.
